# Catalytic Oxidation of Chlorobenzene over Amorphous Manganese-Chromium Catalysts Supported by UiO-66-Derived ZrOx

**DOI:** 10.3390/ma17092103

**Published:** 2024-04-29

**Authors:** Pengfei Zhu, Qiaosen Yuan, Na Li, Zhaoxia Hu, Shouwen Chen

**Affiliations:** School of Biological and Environmental Engineering, Nanjing University of Science & Technology, Nanjing 210094, China; 317102010045@njust.edu.cn (P.Z.); yuanqiaosennjust@163.com (Q.Y.); nli@njust.edu.cn (N.L.); huzhaoxia@njust.edu.cn (Z.H.)

**Keywords:** catalytic oxidation, chlorobenzene, chromium oxides, synergistic effect

## Abstract

The development of efficient catalysts with longevity to remove chlorobenzene is challenging due to Cl poisoning. Herein, a series of Mn-Cr/ZrO_x_ catalysts supported by Zr-based metal-organic framework (UiO-66)-derived ZrO_x_ was prepared and investigated for chlorobenzene (CB) catalytic oxidation. MnCr/ZrO_x_-M prepared via a wet impregnation method presented an amorphous structure, indicating the homogeneous dispersion of Cr and Mn, which improved acid and redox properties. 40Mn7Cr3/ZrO_x_-M exhibited the best catalytic activity for chlorobenzene oxidation with T_90_ of 293 °C, which is mainly due to the strong interaction between manganese and chromium promoted by the large specific surface area of the ZrO_x_ support. Furthermore, 40Mn7Cr3/ZrO_x_-M presented excellent stability for chlorobenzene oxidation.

## 1. Introduction

In recent decades, the emissions of volatile organic compounds (VOCs) have increased dramatically with the rapid development of the industrial economy [1,2]. Among these compounds, chlorinated volatile organic pollutants (CVOCs) are common intermediates and organic solvents in industrial production and waste incineration. Due to their high chemical stability and high toxicity, CVOCs have critically damaged the environment and human health [3,4]. Currently, CVOC treatment technologies include adsorption, photocatalytic degradation, catalytic combustion, ozone oxidation, biological treatment, etc. [5,6]. Catalytic combustion is a very promising technique that can efficiently mineralize CVOCs without producing secondary pollutants [7]. Catalysts play a crucial role in catalytic combustion. A wide range of catalysts are used in this process, including noble metal catalysts, molecular sieve catalysts, and transition metal oxide catalysts [8]. In general, conventional noble metal (e.g., Pt, Pd, and Ru) catalysts exhibit exceptional oxidation capabilities in the destruction of CVOCs. However, their vulnerability to toxic deactivation, sintering, and high prices have strictly limited their practical applications [9,10,11,12,13]. In contrast, transition metal oxide catalysts have attracted high level of concern owing to their resistance to Cl poisoning, low price, and excellent catalytic activity. In addition, the acidity and crystal defects of the transition metal oxides can be customized by doping metal elements, thereby enhancing the selectivity to specific pollutants.

Among non-noble metal catalysts, manganese-based and chromium-based catalysts are very promising owing to their high stability and catalytic oxidation performance [14,15,16]. Wang et al. successfully prepared Fe-Mn oxide catalysts via the oxalate co-precipitation method [17]. The strong interaction between Fe and Mn increased surface Mn^4+^, which improved their surface acidity and redox performance, thereby promoting the degradation of chlorobenzene. Sun et al. prepared Cr-Ti catalysts, analyzed the combustion of dichloroethane and chlorobenzene, and revealed that the increase in the specific surface area and Cr^6+^ content of the catalyst can improve the catalytic activity [18]. Metal-organic framework materials (MOFs) attracted a high level of concern in adsorption treatment, gas separation, and electrochemistry because of their large specific surface area and varied pore structure [19]. In this paper, porous ZrO_x_ derived from a Zr-based metal organic framework (UiO-66) was used as support, providing more active sites and acidic sites, thus enhancing the activity and stability of the catalysts.

Chlorobenzene, a hazardous chemical that can cause irritation, nervous system effects, and potential organ damage, is widely used as an intermediate in chemical production and the petrochemical industry [20,21,22]. Therefore, it is imperative to develop highly efficient catalysts that exhibit exceptional activity as well as long-term stability for practical applications in the catalytic oxidation of chlorobenzene. In this study, which aimed to develop highly efficient catalysts for chlorobenzene elimination, a series MnCrO_x_/ZrO_x_ catalysts were synthesized via a solvothermal method. The crystal structure, surface acidity, redox characteristics, and catalytic combustion performance of the catalysts were studied. We found that the 40Mn7Cr3/ZrO_x_-M presented superior catalytic activity and stability, thereby demonstrating the potential of utilizing ZrOx derived from UiO-66 as a promising support for developing high-performance transition metal catalysts.

## 2. Materials and Methods

### 2.1. Chemicals and Materials

Manganese (II) nitrate solution (A.R. grade, Mn(NO_3_)_2_, 50 wt%), Chromium (III) nitrate (A.R. grade, Cr(NO_3_)_3_·9H_2_O), Terephthalic acid (A.R. grade, C_8_H_6_O_4_), and N,N-dimethylformamide (DMF) (A.R. grade, C_3_H_7_NO) were purchased from Aladdin Biochemical Technology Co.,Ltd., Shanghai, China. Ethanol (A.R. grade, C_2_H_6_O) was purchased from Nanshi Chemical Reagent Co. Ltd., Nanjing, China. Chlorobenzene (A.R. grade, C_6_H_5_Cl), Zirconium tetrachloride (A.R. grade, ZrCl_4_), and Glacial acetic acid (A.R. grade) were purchased from Merrill Chemical Technology Co. Ltd., Shanghai, China. All chemicals were used without further treatment.

### 2.2. Catalyst Preparation

The Zr-based MOF material UiO-66 was prepared via the solvothermal method [23]. Specifically, 18 mmol (2.99 g) of terephthalic acid and 18 mmol (about 4.19 g) of ZrCl_4_ were dissolved in 200 mL N,N-dimethylformamide (DMF), respectively; then, both solutions were mixed together, followed by an addition of 30 mL of glacial acetic acid. After stirring for 30 min, the transparent solution was transferred to a Teflon-lined autoclave, which was then heated at 120 °C for 12 h. The formed solid was centrifuged and then washed 3 times with DMF and absolute ethanol. Finally, the obtained product was dried at 80 °C overnight and denoted as UiO-66. The yield of the UiO-66 was about 80%.

A series of manganese-chromium catalysts was loaded on UiO-66 via the wet impregnation method. Taking 40Mn7Cr3/ZrOx-M as an example, the specific preparation steps used were as follows. First, 5 g UiO-66, 0.979 g Cr(NO_3_)_3_·9H_2_O, and 0.375 g 50 wt% Mn(NO_3_)_2_ solution were added into 100 mL ultrapure water while stirring. After vigorous stirring at 200 rpm for 0.5 h, the solution was then transferred into a rotary evaporator and evaporated at 80 °C under vacuum. The yield of the loaded MnCr/ZrOx-M catalysts was above 90%. The product obtained was dried at 80 °C for 12 h, followed by calcination at 400 °C for 4 h. After grinding, sieving, and tableting (40–60 meshes), a 40Mn7Cr3/ZrOx-M catalyst was obtained. ZrOx-M was also prepared with the same procedure without the addition of Cr(NO_3_)_3_·9H_2_O and Mn(NO_3_)_2_ solutions.

### 2.3. Catalyst Characterization

The catalysts were systematically characterized using various techniques, including X-ray diffraction (XRD), scanning electron microscopy (SEM), energy-dispersive spectroscopy (EDS), N_2_ adsorption–desorption analysis, X-ray photoelectron spectroscopy (XPS), H_2_ temperature-programmed reduction (H_2_-TPR), and temperature-programmed desorption of ammonia (NH_3_-TPD).

The samples were analyzed via powder X-ray diffraction technology using a BRUKER D8ADVANCE powder diffractometer. The instrument was equipped with Cu Kα radiation (λ = 1.54178 Å) and operated at 40 kV and 40 mA. The diffraction patterns were recorded within the range of 5 to 90°, employing a scan speed of 10°/min. Scanning electron microscopy (SEM) images were captured using an FEI Quanta 250FEG electron microscope operating at an acceleration voltage ranging from 3 to 15 kV. The elemental distribution of the samples was analyzed through Oxford X-Max energy-dispersive spectroscopy (EDS). The BET surface area (S_BET_) and pore volume (Vpore) of the catalysts were measured at 77 K using a Micromeritics ASAP 2020 automatic specific surface analyzer, followed by pretreatment under vacuum at 200 °C for 2 h. The Brunauer–Emmett–Teller equation was employed to obtain the specific surface area, while the Barrett–Joyner–Halenda (BJH) method was utilized to measure the pore size distribution. The XPS analysis was conducted using a Thermo ESCALAB 250XI spectrometer (Thermo Fisher Scientific, Waltham, MA, USA), which was equipped with AlK radiation (1486.6 eV) as the excitation source.

The H_2_-TPR experiment was conducted using conventional apparatus equipped with a TCD detector. The sample (50 mg) underwent pretreatment at 400 °C for 1 h under a N_2_ flow (30 mL/min), followed by cooling to room temperature. Subsequently, the samples were heated in a 5% H_2_/Ar (N_2_) flow (30 mL/min) at a heating rate of 5 °C/min from 50 to 800 °C. To calibrate the amount of H_2_ consumption, we utilized complete reduction of a standard CuO sample weighing 50 mg. For NH_3_-TPD analysis, we employed the same apparatus as used for H_2_-TPR. Prior to analysis, each sample (100 mg) was pretreated at 400 °C for an hour with N_2_ gas and then cooled down to 50 °C. Next, the samples were exposed to a NH_3_ flow (dried gaseous ammonia, 20 mL/min) for at least one hour to achieve saturation of adsorption. Subsequently, the saturated sample was flushed with N_2_ gas (30 mL/min) at the same temperature for half an hour in order to eliminate physically adsorbed NH_3_. The desorption process involved heating the samples in N_2_ flow (30 mL/min), gradually increasing the temperature from 50 to 800 °C at a rate of 5 °C per minute.

### 2.4. Catalytic Test

**Figure 1:**
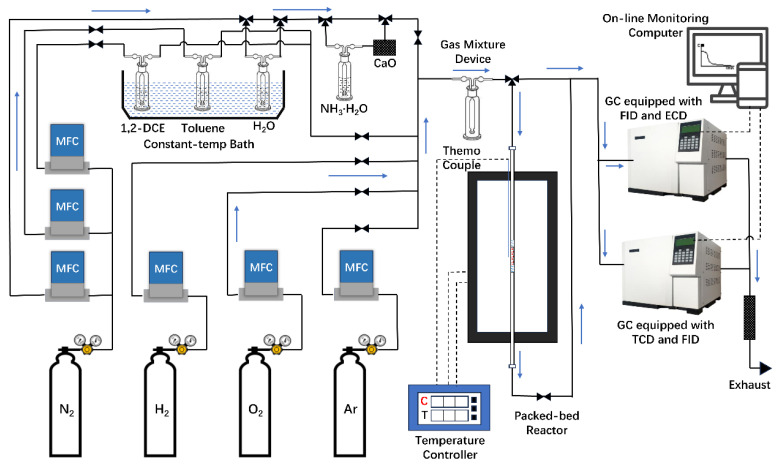
Catalyst performance evaluation system.

The performance of catalytic combustion for chlorobenzene was evaluated in a fixed-bed reactor consisting of a quartz tube (i.d. = 4 mm) under standard atmospheric pressure. In each experiment, 100 mg of catalysts (40–60 mesh) was immobilized using quartz wool, resulting in a gas hourly space velocity (GHSV) of 20,000 mL·g^−1^ h^−1^ and a total flow rate of 33.33 mL·min^−1^. The liquid reactant (chlorobenzene) was introduced into the feed stream by passing dried air through a saturator maintained at a temperature of 6 °C. The feed stream was then diluted with dried air generating a feeding flow containing 1000 ppm reactant and 21% O_2_/79% N_2_. Flow rates were regulated using online mass flowmeters. Concentrations of CB and chlorinated byproducts were determined via analysis on an online gas chromatograph (GC3420A, Beifen Ruili Co., Ltd., Beijing, China) equipped with both flame ionization detector (FID) and electron capture detector (ECD) for quantitative assessment of organic compounds. Furthermore, the outlet CO_2_ underwent reduction by hydrogen within a methanation furnace before being detected by the same chromatograph system. A K-type thermocouple was inserted into the catalyst to monitor its temperature during the catalytic performance test. Data of catalytic oxidation tests were collected after achieving steady state conditions for at least thirty minutes. The detailed structure of the catalytic test system is shown in Figure 1.

The CB conversion rate ($X_{CB}$, %), *CO*_2_ *yield* (${CO}_{2}yield$, %) and organic byproduct yield ($Y_{b}$, %) were calculated as follows:

(1) $$X_{CB}=\frac{{\left(CB\right)}_{in}-{\left(CB\right)}_{out}}{{\left(CB\right)}_{in}}\times 100\%,$$

(2) $${CO}_{2}yield=\frac{({{CO}_{2})}_{out}}{6\times {\left(CB\right)}_{in}}\times 100\%,$$

(3) $$Y_{b}=X_{CB}-{CO}_{2}yield,$$

where ${\left(CB\right)}_{in}$ and$\;{\left(CB\right)}_{out}$ are the inlet and outlet concentrations of chlorobenzene, respectively. $({{CO}_{2})}_{out}$ is the outlet concentrations of CO_2_.

The reaction rate of 1,2-dichloroethane was calculated as follows:

(4) $$r=\frac{Q\times X_{CB}}{m}$$

where r is the reaction rate (mol·g^−1^·s^−1^), *Q* is the molar flow of chlorobenzene (mol·s^−1^), and *m* is the mass of the catalyst (g).

The stability test for chlorobenzene was performed continuously at 290–320 °C for 100 h under the same conditions as the catalytic combustion test, unless otherwise indicated.

## 3. Results and Discussions

### 3.1. Catalytic Performance for Chlorobenzene Oxidation

**Figure 2:**
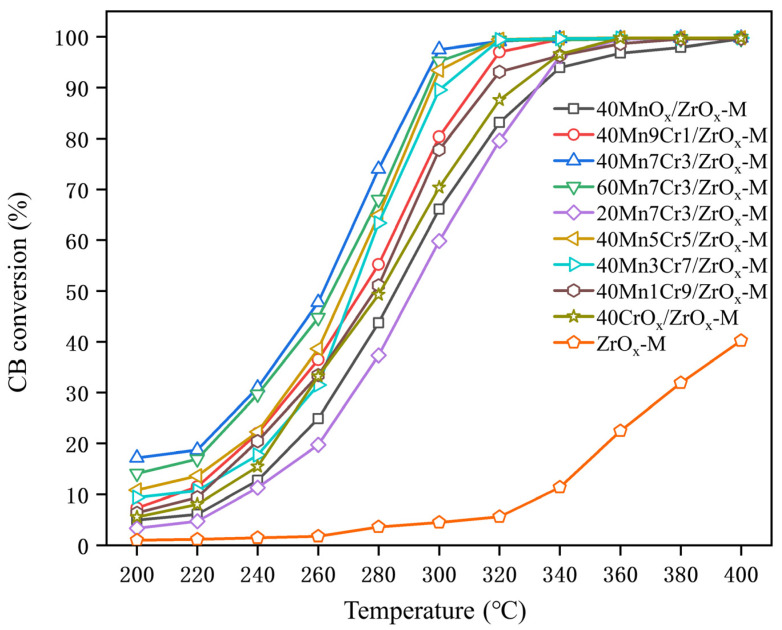
The chlorobenzene conversion curves over MnCr/ZrOx-M catalysts; gas composition: 1000 ppm chlorobenzene; GHSV = 20,000 h^−1^; catalyst amount: 100 mg.

Catalytic oxidation of chlorobenzene was tested on the prepared catalysts, and the light-off curves are shown in Figure 2. The temperatures required for achieving 50% and 90% conversion of chlorobenzene (T_50_ and T_90_) are listed in Table 1. The sequence in catalytic activity for CB oxidation at SV = 20,000 mL·g^−1^·h^−1^ was 40Mn7Cr3/ZrO_x_-M (T_90_ = 293 °C) > 60Mn7Cr3/ZrO_x_-M (T_90_ = 295 °C) > 40Mn5Cr5/ZrO_x_-M (T_90_ = 296 °C) > 40Mn3Cr7/ZrO_x_-M (T_90_ = 301 °C) > 40Mn9Cr1/ZrO_x_-M (T_90_ = 311 °C) > 40Mn1Cr9/ZrO_x_-M (T_90_ = 315 °C) > 40CrOx/ZrO_x_-M (T_90_ = 322 °C) > 20Mn7Cr3/ZrO_x_-M (T_90_ = 330 °C) > 40MnO_x_/ZrO_x_-M (T_90_ = 331 °C) > ZrO_x_-M (T_90_ > 400 °C). Overall, MnCr/ZrO_x_-M catalysts demonstrated excellent performance in the oxidation of chlorobenzene. It is worth noting that the MnCr/ZrO_x_-M catalysts exhibited significantly enhanced activity compared to pristine ZrO_x_-M. The addition of Cr into MnO_x_ notably improved the performance of chlorobenzene oxidation, confirming the high activity of Cr-Mn oxides for CB oxidation. However, excessive Cr doping resulted in a decrease in the amount of acid sites and active oxygen species on the catalyst, leading to reduced catalytic activity. Furthermore, the loading rate of Mn-Cr oxides onto the ZrO_x_ substrate played a crucial role in determining the performance of MnCr/ZrO_x_-M. Specifically, 40Mn7Cr3/ZrO_x_-M exhibited the most favorable performance. A loading rate of 20% resulted in a lower proportion of Mn^4+^ and Cr^6+^, potentially leading to a decrease in active sites. Conversely, an excessive loading rate of 60% led to a reduction in the specific surface area, indicating a blocked mesoporous structure of ZrO_x_-M. In summary, the Mn/Cr ratio of 7:3 and a loading rate of 40% promote the interaction between Mn, Cr, and ZrO_x_ substrates, leading to an augmentation in surface active oxygen and acidic sites of the catalyst. Consequently, this enhances the low-temperature catalytic activity of the catalyst towards chlorobenzene.

### 3.2. Durability and Stability

**Figure 3:**
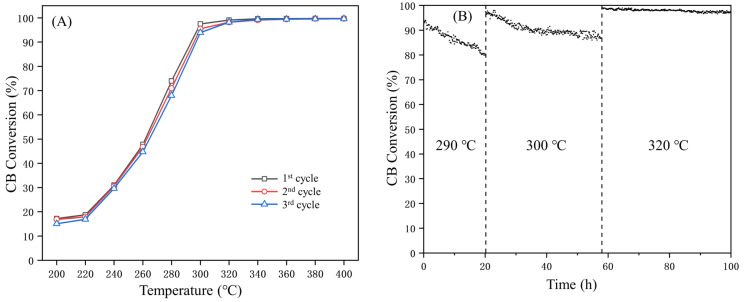
The three-cycle conversion curves over 40Mn7Cr3/ZrOx-M (**A**) and the stability of the 40Mn7Cr3/ZrOx-M catalyst (**B**); gas composition: 1000 ppm chlorobenzene; GHSV = 20,000 h^−1^; catalyst amount: 100 mg.

In practical applications, catalyst poisoning significantly reduces the operational lifespan of the catalyst and substantially increases the operation cost of the catalytic combustion method [7]. Therefore, the durability and stability of catalysts are equally crucial to their catalytic ability in engineering applications. As depicted in Figure 3A, the catalytic activity remained nearly unchanged after a three-cycle test, indicating excellent durability of the catalyst. Moreover, no significant decline in conversion rates was observed after 100 h of the on-stream test. As depicted in Figure 3B, the conversion of chlorobenzene gradually decreased to 80% at 290 °C within the initial 20 h period. This decline could potentially be attributed to the accumulation of chlorine species on the surface of catalyst, resulting in the obstruction of active sites. After being heated to 300 °C, the conversion rate exhibited a significant increase and subsequently stabilized at approximately 90%. After reaching a temperature of 320 °C, the accumulated chlorine and carbon species on the catalyst surface desorb, exposing the active sites again. After reaching a temperature of 320 °C, the accumulated chlorine and carbon species were decomposed and the blocked active sites were re-exposed. As a result, the conversion rate was restored and consistently maintained at approximately 99%, with negligible variations observed over a period of 40 h. The ZrO_x_ substrate provided a significant number of acidic sites, leading to enhanced stability and a prolonged lifespan. These improvements make the developed catalyst more practical for diverse industrial applications.

### 3.3. Crystal Structure and Texture Properties

**Figure 4:**
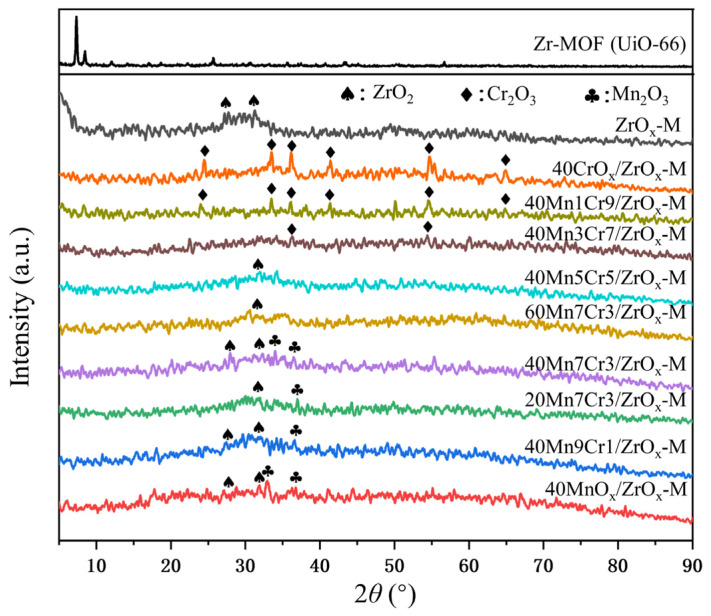
XRD patterns of the Mn-Cr/ZrO_x_-M catalysts and Zr-MOF.

XRD patterns of Mn-Cr/ZrOx-M series catalysts and Zr-MOF are shown in Figure 4. The pattern of Zr-MOF (UiO-66) synthesized via the solvothermal method is consistent with the reported literature [28]. After calcination, the typical characteristics peaks of UiO-66 disappeared and obtained ZrO_x_-M catalysts derived from UiO-66 exhibited weak diffraction peaks of orthorhombic phase ZrO_2_ (PDF#41-0017), suggesting that UiO-66 decomposed into amorphous ZrO_2_. The EDS results of ZrOx-M also proved that organic ligands complete decomposed, leaving only a minimal amount of residual carbon species. Several characteristic peaks at 24.5°, 33.6°, 36.2°, 41.5°, 54.9°, and 65.1° were detected on 40CrOx/ZrOx-M, and they were assigned to the rhombohedral phase of Cr_2_O_3_ (PDF#06-0504) [29]. However, no peaks corresponding to ZrO_2_ were detected, suggesting that the Cr species were incorporated into the lattice of ZrO_2_ rather than physically loaded onto its surface. This incorporation led to a further decrease in the crystallinity of ZrO_2_. In contrast, diffraction peaks corresponding to both cubic phase Mn_2_O_3_ (PDF#10-0069) [30] and ZrO_2_ were observed on the 40MnO_x_/ZrO_x_-M, suggesting that MnO_2_ was simply loaded on the surface of ZrO_2_. After the introduction of Mn into 40CrOx/ZrOx-M, the characteristic peaks of Cr_2_O_3_ were significantly weakened, indicating that Mn infiltrated the lattice of Cr_2_O_3_ and formed an amorphous structure. No diffraction peaks for Mn5Cr5, Mn7Cr3 and Mn9Cr1 were detected, suggesting that Mn and Cr species were highly dispersed on the ZrOx support. The amorphous structure demonstrated strong interactions between manganese and chromium, which promoted the generation of lattice defects and the formation of oxygen vacancies, thus improving catalytic performance [5].

**Figure 5:**
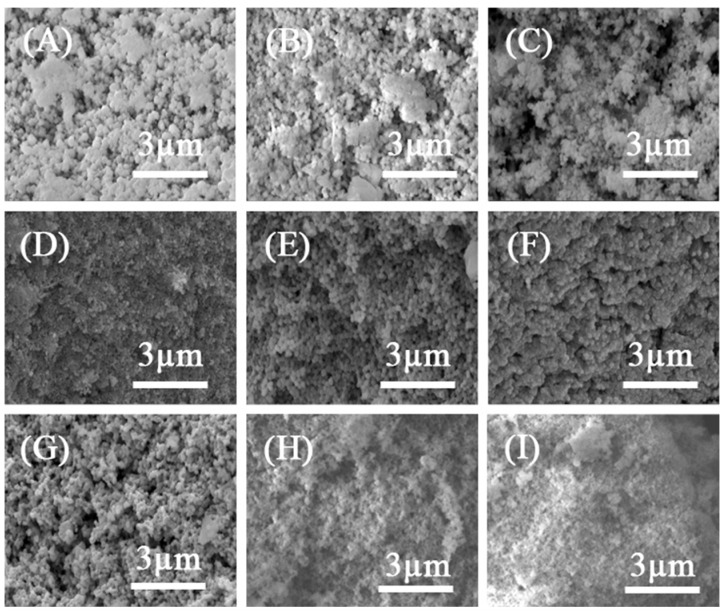
SEM images (**A**–**I**) of the Mn-Cr/ZrO_x_-M catalysts: (**A**) 40MnO_x_/ZrO_x_-M; (**B**) 40Mn9Cr1/ZrO_x_-M; (**C**) 40Mn7Cr3/ZrO_x_-M; (**D**) 60Mn7Cr3/ZrO_x_-M; (**E**) 40Mn5Cr5/ZrO_x_-M; (**F**) 40Mn3Cr7/ZrO_x_-M; (**G**) 40Mn1Cr9/ZrO_x_-M; (**H**) 40CrOx/ZrO_x_-M; (**I**) used 40Mn7Cr3/ZrO_x_-M.

**Figure 6:**
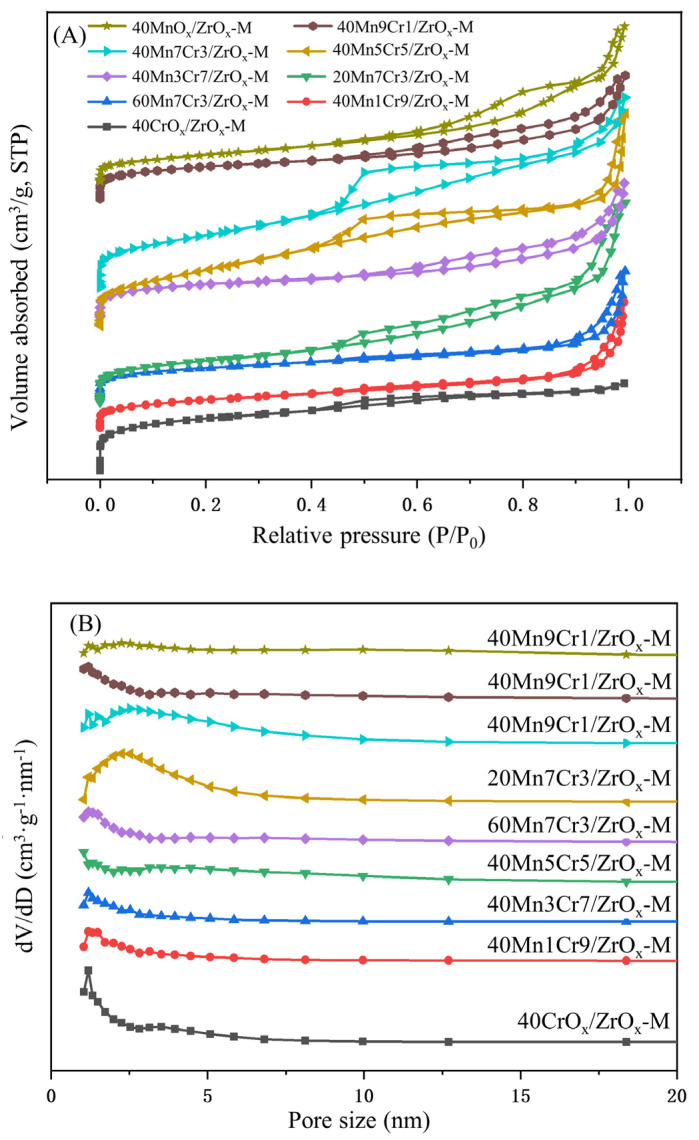
N_2_ adsorption–desorption isotherms (**A**) and pore size distribution curves of catalysts (**B**).

The SEM images of Mn-Cr/ZrO_x_-M catalysts are shown in Figure 5. All catalysts presented an irregular clusters structure, resulting in a large specific surface area. This was also confirmed by the N_2_ adsorption–desorption results (Figure 6). With the gradual increase in Cr content, the agglomerated particles were transformed into finer particles on the catalyst’s surface. However, when the Cr proportion exceeded that of Mn, an accumulation of fine particles occurred on the surface of the ZrO_x_-M support, leading to pore blockage and a significant reduction in specific surface area. This phenomenon is corroborates with the N_2_ adsorption–desorption results. Furthermore, it can be observed that the 60Mn7Cr3/ZrO_x_-M catalyst exhibited minimal surface porosity, which could be attributed to excessive loading. After the catalytic stability test, the surface porosity of 40Mn7Cr3/ZrOx-M catalyst was slightly decreased, while its fine granular structure remained intact. This result demonstrates the remarkable stability of the prepared catalysts. The utilization of ZrO_2_ derived from UiO-66 as a support, in combination with appropriate proportions of Mn and Cr loading, facilitates structural and morphological modifications of catalysts.

To investigate the texture characteristics of the as-prepared catalysts, nitrogen adsorption–desorption measurements were conducted. The isotherms for adsorption–desorption of nitrogen and the distribution of pore sizes (using BJH method) for Mn-Cr/ZrOx-M series catalysts are illustrated in Figure 6A and Figure 6B, respectively. The corresponding results, including the specific surface area (S_BET_), average pore size (D_p_), and total pore volume (V_p_) of the catalysts, are summarized in Table 2. All catalysts presented a typical IV adsorption isotherm with H_3_-type hysteresis loops, revealing the mesoporous structure of catalysts, which can be further confirmed by the pore size distribution results [31]. Compared to the conventional sol–gel method and co-precipitation method, catalysts utilizing a porous ZrO_2_ substrate exhibited a significantly enhanced specific surface area, thereby facilitating the exposure of acid sites and active sites [32]. The CrO_x_ particles exhibited a smaller size compared to MnO_x_, allowing them to infiltrate into the mesoporous channels of MnO_x_ and create additional pore structures [33,34]. However, an excessive amount of CrOx caused a blockage in the mesopores channel of MnOx, leading to a significant decrease in the specific surface area. As the Cr content increased, there was initially an increase in the specific surface area observed for the catalysts, followed by a subsequent reduction. This observation is consistent with the findings from scanning electron microscopy (SEM) analysis. Among all the catalysts, 40Mn7Cr3/ZrOx-M exhibited superior specific surface area and pore volume. This resulted in reduced diffusion resistance between chlorobenzene molecules and the active sites of the catalysts, ultimately enhancing their catalytic performance.

### 3.4. Chemical States, Redox, and Acidity Properties

**Figure 7:**
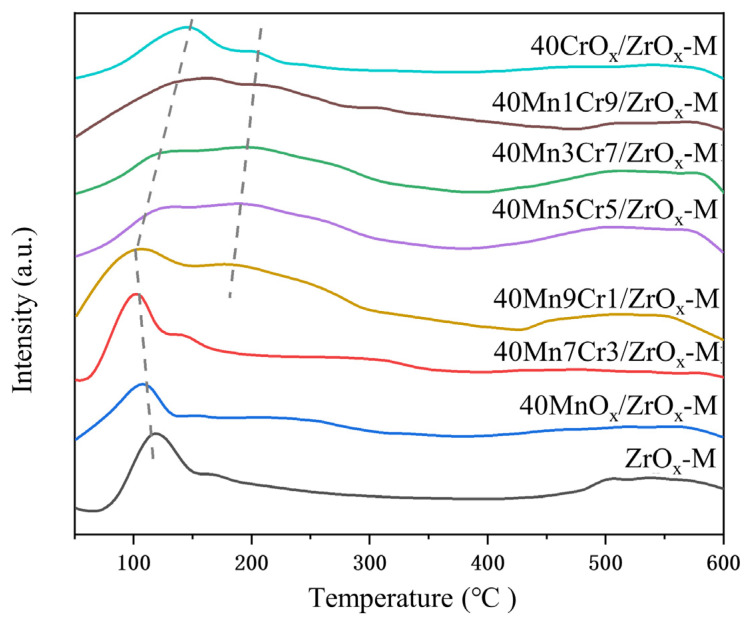
NH_3_-TPD profiles of the Mn-Cr/ZrO_x_-M catalysts.

The acidic sites of the catalysts were studied via NH_3_-TPD, and the corresponding results are presented in Figure 7. It has been proven that NH_3_ desorption temperature reflects the acidity of a catalyst’s surface and that the desorption peak area reveals the quantity of acid sites [35]. NH_3_ desorption curves of the catalysts presented a wide ammonia desorption peak, which could be divided into weak acidic site peaks (50–200 °C) and strong acidic site peaks (200–400 °C) [36,37]. Recent studies have demonstrated that the presence of weak acid sites enhances the adsorption of chlorobenzene, while strong acid sites promote the desorption of chlorine intermediates [38,39,40]. Both types of acid sites contribute to increased catalytic performance. Figure 7 demonstrates the substantial occurrence of weak acid sites in all Mn-Cr/ZrO_x_-M catalysts. The desorption peaks observed at temperatures ranging from 50 to 200 °C primarily originated from the ZrO_x_-M support, which was closely related to the high specific surface area of the catalysts [41]. The addition of Cr significantly increased the amount of weak acid sites in Mn1Cr9 to the Mn7Cr3 samples, which promoted the catalytic oxidation of chlorobenzene [42]. The strong interaction between well-dispersed Mn and Cr on the catalyst surface promoted the formation of surface hydroxyl groups, thereby forming strong acid sites corresponding to the broad peak above 200 °C [26]. Cr notably increased the high-temperature peak area of the profiles, suggesting that the catalysts had abundant strong acid sites, which promoted the deep oxidation of CVOCs. The moderate doping of Cr leads to a shift in the NH_3_ desorption peak towards higher temperatures, thereby increasing the acidity of the catalysts. This enhancement promotes the deep oxidation of CVOCs. However, an excessive doping rate of Cr may weaken the interaction between Mn and Cr, leading to a significant reduction in the number of strong acid sites and consequently reducing catalytic performance.

**Figure 8:**
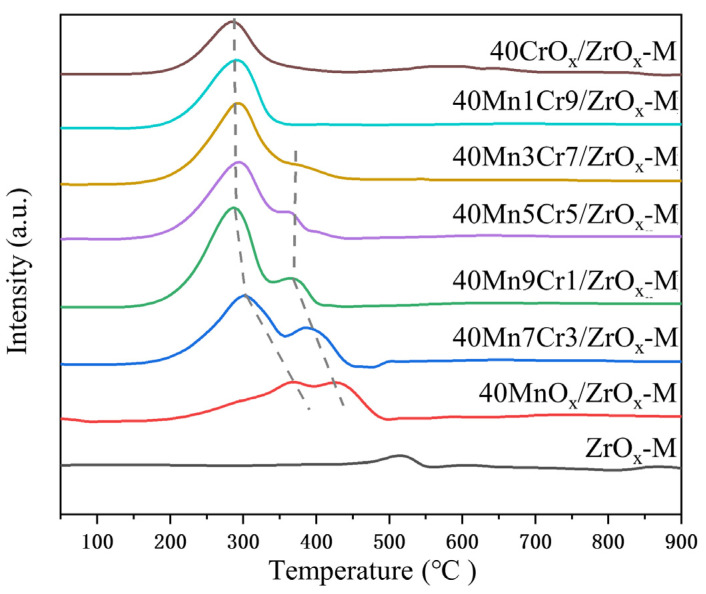
H_2_-TPR profiles of the Mn-Cr/ZrOx-M catalysts.

The redox property of Mn-Cr/ZrO_x_-M series catalysts was investigated via H_2_-TPR, and their profiles are shown in Figure 8. In general, the valence state and the quantity of active oxygen are crucial to redox performance [43]. For ZrO_x_-M, a weaker reduction peak was observed at around 520 °C, suggesting that the pristine ZrO_x_-M support exhibited relatively modest reducing performance. The profile of 40MnOx/ZrO_x_ displayed two reduction peaks at 375 °C and 440 °C, corresponding to a reduction in Mn^4+^ and Mn^3+^, respectively [44]. Generally speaking, the reduction in MnO_x_-based catalysts, which undergo successive processes of MnO_2_ → Mn_3_O_4/_Mn_2_O_3_ → MnO, corresponds to low-temperature reduction peaks (<400 °C) and high-temperature reduction peaks, respectively [45]. The 40CrO_x_/ZrO_x_-M catalyst presented a significant reduction peak at 300 °C, corresponding to the reduction in Cr^6+^ to Cr^3+^ [46]. After doping Cr into 40MnOx/ZrO_x_-M, it was observed that the reduction peaks of Mn^4+^ and Cr^6+^ merged to form a larger peak. This suggests that there was a strong interaction between MnO_x_ and CrO_x_, which enhanced the mobility of the surface lattice oxygen and subsequently improved catalytic activity [47]. Moreover, the addition of Cr led to increased reduction peak areas that shifted towards lower temperatures, thereby enhancing the redox performance at low temperatures [26]. However, excessive Cr doping resulted in the absence of a reduction peak at 375 °C, indicating the weaker redox ability of Mn3Cr7 and Mn1Cr9. The 40Mn7Cr3/ZrO_x_-M catalyst exhibited the largest H_2_ reduction peak at the lowest temperature, suggesting excellent redox activity. These findings are consistent with the results of the catalytic performance test.

**Figure 9:**
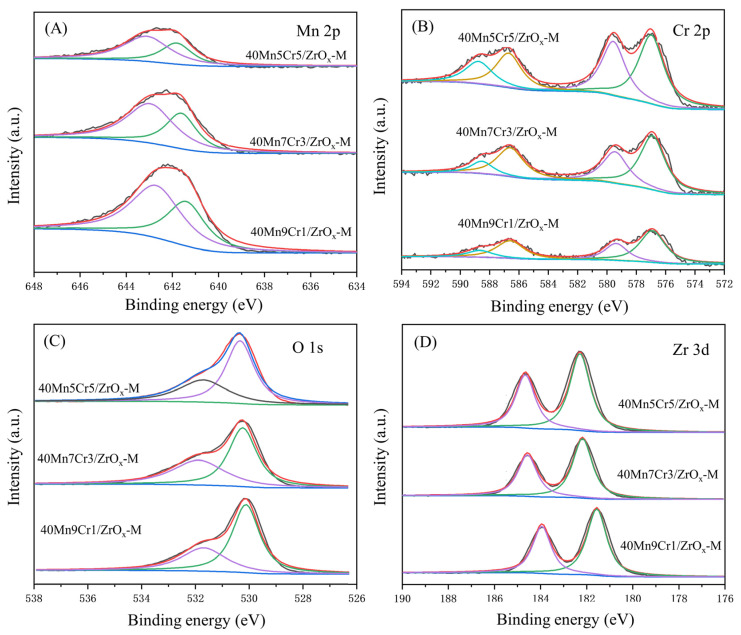
XPS spectra of the MnCr/ZrOx-M catalysts: (**A**) Mn 2p; (**B**) Cr 2p; (**C**) O 1s; (**D**) Zr 3d.

Chemical states and oxidation states of the catalysts were studied via Mn 2p, Cr 2p, and Zr 3d XPS spectra, the patterns of which are shown in Figure 9, and the quantitative results are summarized in Table 1. The Mn 2p spectra (Figure 9A) exhibited two binding energy peaks in the range of 642.9–643.2 eV and 641.4–641.9 eV regions, which were attributed to Mn^4+^ and Mn^3+^, respectively. This demonstrates the coexistence of Mn^4+^ and Mn^3+^ on the catalyst’s surface. However, Mn^4+^ characteristic peaks were absent in the XRD patterns, indicating that Mn^4+^ species were uniformly dispersed on the surface of the catalyst with an amorphous structure. With the addition of Cr, the Mn 2P spectra shifted towards higher-binding-energy regions, indicating an increased electron cloud density in Mn^4+^ and Mn^3+^ due to the interaction between Cr and Mn [48]. In addition, the peak position of Zr also shifted to higher-binding-energy regions with the doping of Cr, which revealed a strong interaction between the ZrO_x_ support and Mn-Cr oxides. According to the results presented in Table 2, the proportion of Mn^4+^ noticeably increased following Cr doping due to the strong interaction between the two elements. Moreover, higher-valence Mn species are more favorable for promoting oxidation reactions and facilitating the generation of oxygen vacancies [49]. For the Cr 2p spectrum, the binding energy peaks at 588.4–588.8 eV and 579.4–579.6 eV regions were assigned to Cr^6+^, and the binding energy peaks at 586.6–586.7 eV and 576.9–577.0 eV regions were attributed to Cr^3+^. These results suggest that Cr^6+^ and Cr^3+^ coexist on the catalyst’s surface. The absence of characteristic peaks for CrO_x_ in XRD patterns indicates that Cr^6+^ is uniformly dispersed on the catalyst’s amorphous surface structure, which enhances the deep oxidation of chlorobenzene [18]. Broad and asymmetric peaks were observed in the O 1s spectra. The peak at an electronic binding energy of 531.6–351.8 eV was assigned to adsorbed oxygen (O_ads_), while the peak at 530.1–530.4 eV was attributed to lattice oxygen (O_latt_). Previous studies have demonstrated that O_ads_ is the most active oxygen species, promoting the transportation and transformation of oxygen species [50,51,52]. Therefore, a higher content of O_ads_ improves catalytic combustion performance. According to the peak area results in Table 1, it can be seen that the 40Mn7Cr3/ZrO_x_-M exhibited the largest ratio of O_ads_/O_latt_, which was consistent with the catalytic performance results.

**Figure 10:**
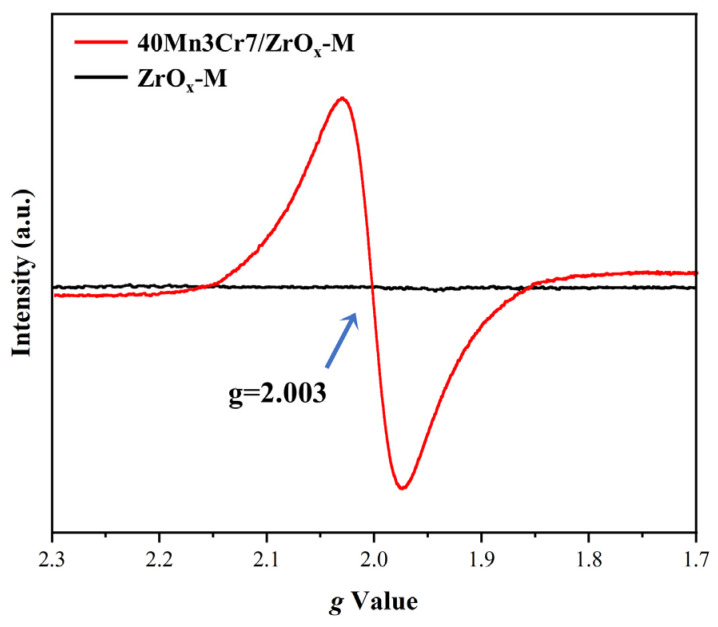
EPR profiles of the Mn-Cr/ZrOx-M catalysts.

As shown in Figure 10, the EPR profile of the Mn-Cr/ZrOx-M catalysts presented a signal-centered g = 2.003, which could be attributed to the electrons trapped in oxygen vacancies [51]. In addition, the EPR signal intensity of 40Mn7Cr3/ZrO_x_-M was significantly higher than that of ZrO_x_-M. This suggests that the loaded MnCrO_x_ can offer abundant oxygen vacancies.

## 4. Conclusions

In this study, a series of Mn-Cr/ZrOx-M catalysts with different proportions of manganese-chromium oxides were synthesized using ZrO_x_ derived from UiO-66 as a substrate. After the loading of the Mn-Cr oxide, the catalytic performance of the MnCr/ZrO_x_-M catalyst was significantly enhanced compared to that of pristine ZrOx-M. Among the MnCr/ZrO_x_-M catalysts, the 40Mn7Cr3/ZrO_x_-M with mesoporous-structured irregular clusters achieved superior activity with the T_90_ of 293 °C for the catalytic oxidation of chlorobenzene at the WHSV of 20,000 h^−1^. Furthermore, the 40Mn7Cr3/ZrO_x_-M catalyst exhibited developed catalytic activity and durability, making it a promising candidate for practical applications. This exceptional catalytic performance can be mainly attributed to its remarkable oxidizing capability and the abundant presence of active oxygen species on its surface. These characteristics are a direct consequence of the strong interaction between Mn and Cr, resulting in the formation of amorphous MnCrO_x_ oxides. Moreover, the mesoporous structure of the ZrO_x_-M substrate promotes the exposure of acid sites and active sites, thereby enhancing both the low-temperatures catalytic performance and lifespan of the catalyst. In summary, this study provides valuable insights into the effective application of additional components to enhance the production of reactive oxygen species for the purpose of CVOCs’ catalytic decomposition.

## Figures and Tables

**Table 1:** Comparison with existing research.

Catalyst	CVOCs	Concentration	Gaseous Hourly Space Velocity	T_90_ (°C)	References
MnO_x_	Chlorobenzene	500 ppm	22,500 mL·g^−1^·h^−1^	301	[24]
Ru/LaMnO_x_	Chlorobenzene	500 ppm	20,000 mL·g^−1^·h^−1^	356	[25]
Pt-110Mn	Chlorobenzene	1000 ppm	30,000 mL·g^−1^·h^−1^	290	[26]
La_0.9_Ce_0.1_CoO_3_	Chlorobenzene	1000 ppm	60,000 mL·g^−1^·h^−1^	422	[27]
40Mn7Cr3/ZrO_x_-M	Chlorobenzene	1000 ppm	20,000 mL·g^−1^·h^−1^	293	This work

**Table 2:** Characterization results and catalytic performance of the catalysts.

Catalyst	S_BET_ (m^2^/g) ^a^	D_P_ (nm) ^b^	V_P_ (cm^3^/g) ^c^	Mn^4+^/Mn^3+^	Cr^6+^/Cr^3+^	O_ads_/O_latt_	T_50_ (°C)	T_90_ (°C)
40MnO_x_/ZrO_x_-M	113.1	9.7	0.27	-	-	-	285	331
40Mn9Cr1/ZrO_x_-M	136.6	6.3	0.22	1.53	0.43	0.56	274	311
40Mn7Cr3/ZrO_x_-M	231.4	5.8	0.34	1.68	0.50	0.79	262	293
20Mn7Cr3/ZrO_x_-M	238.9	5.9	0.35	-	-	-	290	330
60Mn7Cr3/ZrO_x_-M	140.5	6.5	0.23	-	-	-	264	295
40Mn5Cr5/ZrO_x_-M	172.0	8.3	0.34	1.78	0.71	0.59	269	296
40Mn3Cr7/ZrO_x_-M	110.9	7.4	0.20	-	-	-	271	301
40Mn1Cr9/ZrO_x_-M	121.5	7.0	0.21	-	-	-	279	315
40CrO_x_/ZrO_x_-M	218.4	2.9	0.16	-	-	-	280	322
ZrO_x_-M	-	-	-	-	-	-	>400	>400

^a^ BET specific surface area. ^b^ Total pore volume estimated at *p*/*p*_0_ = 0.99. ^c^ BJH pore diameter calculated from the absorption branch.

## Data Availability

Data will be made available on request.
